# Supplementary figures and images for: Challenges and progress in RNA velocity: Comparative analysis across multiple biological contexts
Source: PLoS Comput Biol. 2026 Jun 1;22(6):e1014303. doi: 10.1371/journal.pcbi.1014303 (PMC13252846; doi:10.1371/journal.pcbi.1014303)

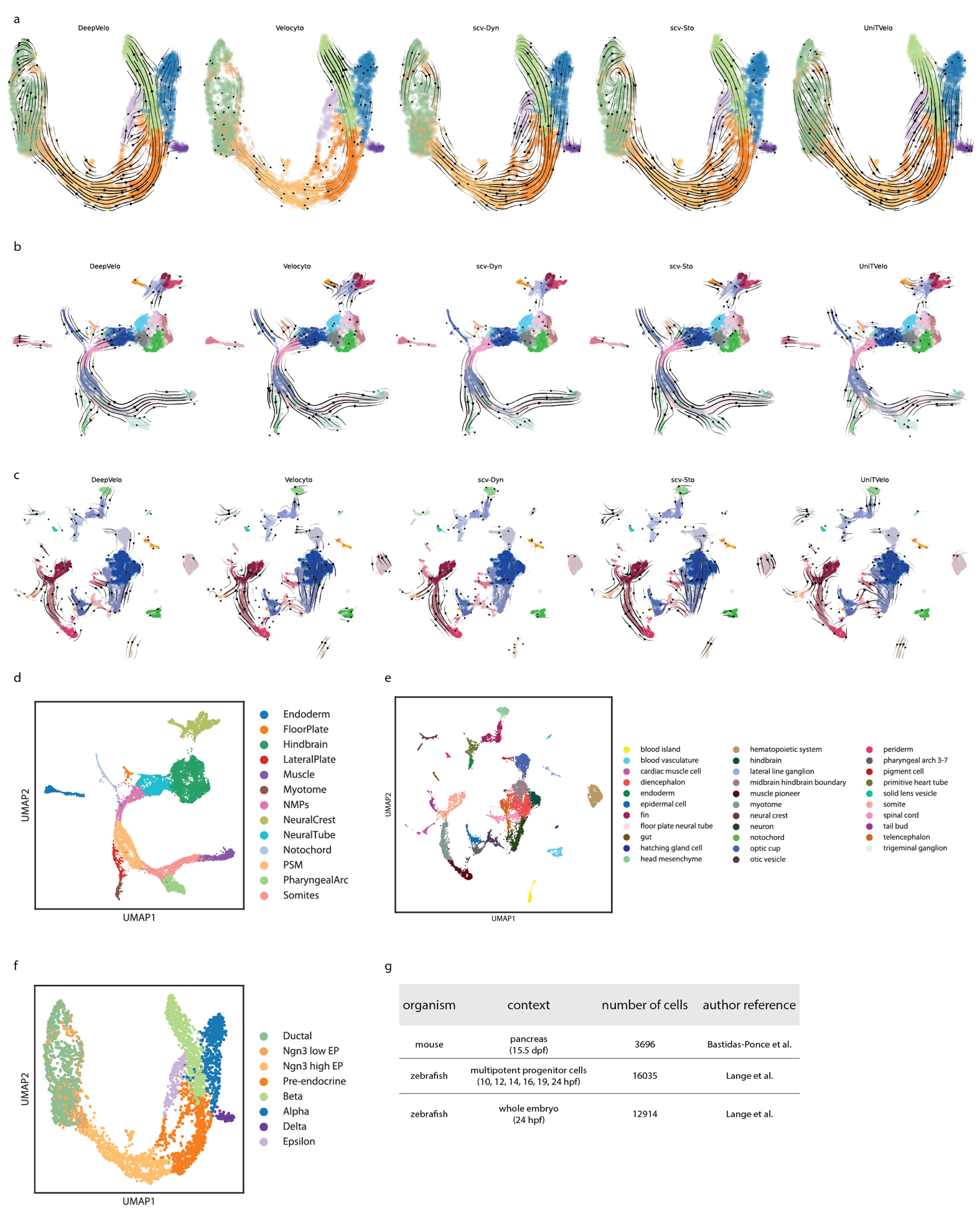

Supplement: S1 Fig — a. RNA velocity UMAP projections for five methods, implemented in the pancreas dataset. b. RNA velocity UMAP projections for five methods, implemented in the zebrafish NMP (ZF NMP) dataset. c. RNA velocity UMAP projections for five methods, implemented in the zebrafish full embryo 24 hours post fertilization (ZF embryo 24hpf) dataset. d. ZF NMP UMAP colored by cell type annotations. e. ZF embryo 24hpf UMAP colored by cell type annotations. f. Pancreas UMAP colored by cell type annotations. g. Table of datasets used in the paper, including information about the organism, biological context, number of cells, and author reference. (TIF) [file pcbi.1014303.s001.tif]

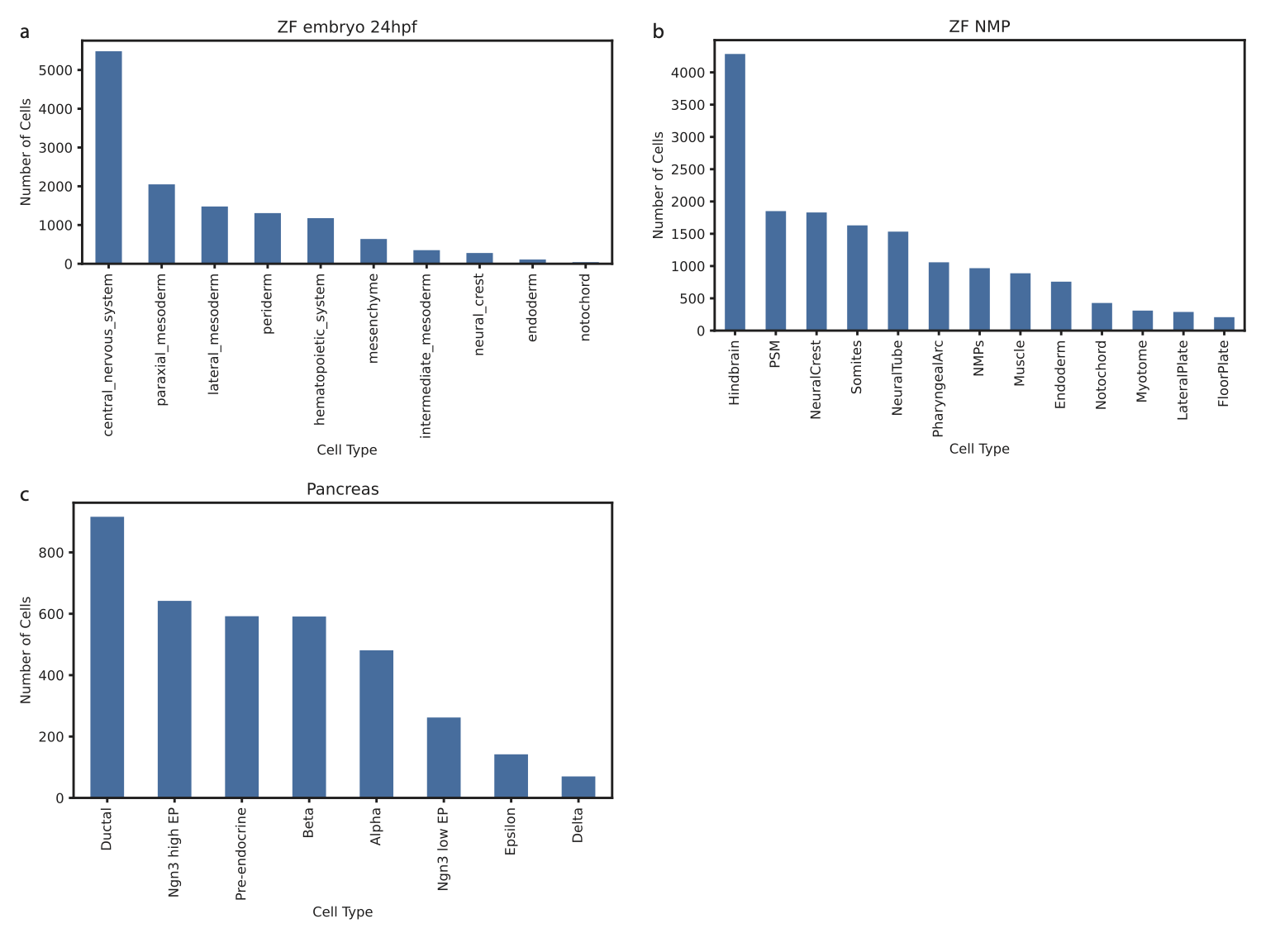

Supplement: S2 Fig — Number of cells per cell type for the ZF 24hpf whole-embryo dataset. b. Number of cells per cell type for the ZF NMP dataset. c. Number of cells per cell type for the pancreas dataset. (TIF) [file pcbi.1014303.s002.tiff]

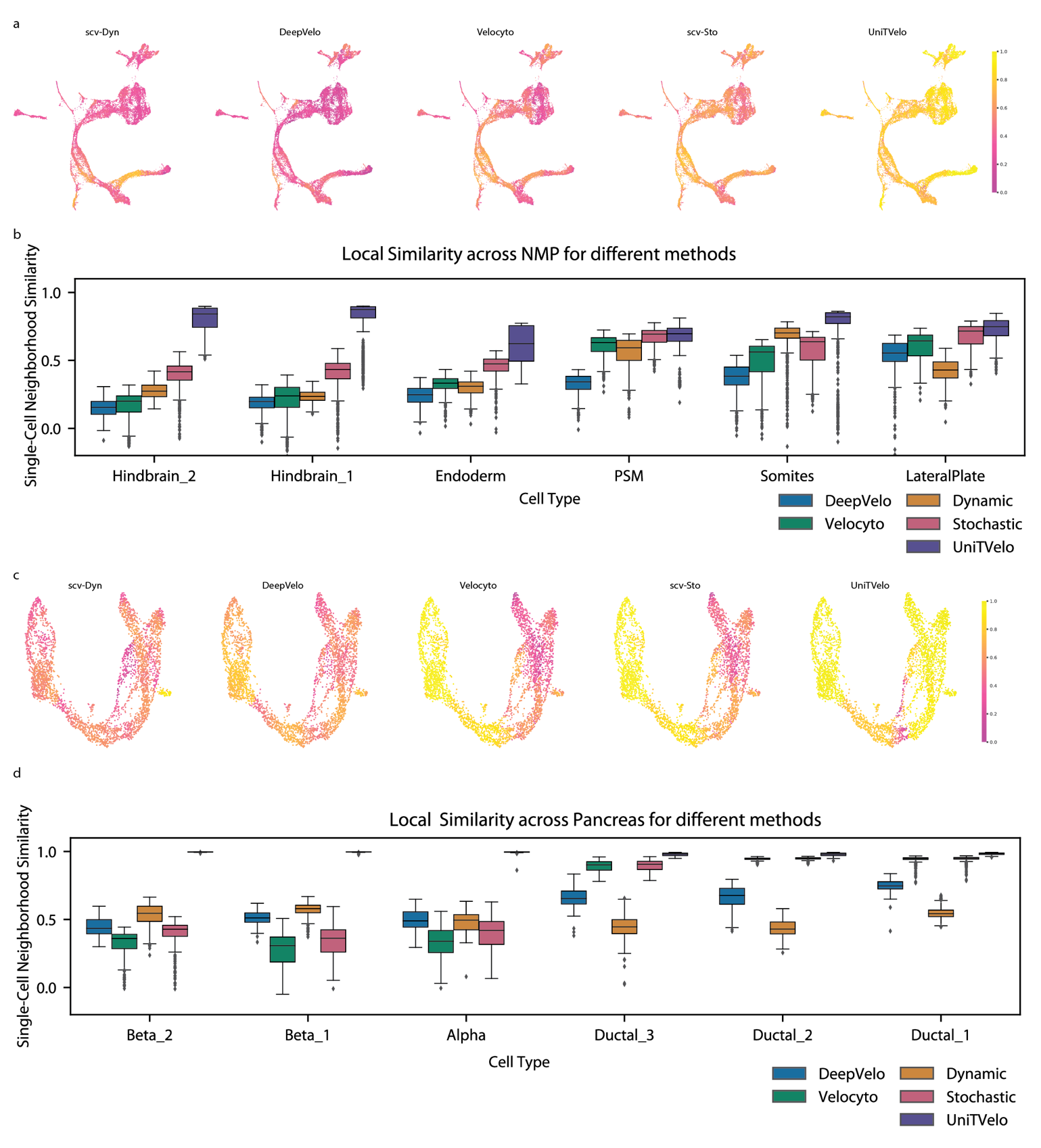

Supplement: S3 Fig — UMAP embeddings for ZF NMP dataset colored by the single-cell local consistency for each RNA velocity method. b. Local consistency distributions for the three top and bottom cell types from the ZF NMP dataset, as ranked by average local consistency. c. UMAP embeddings for pancreas dataset colored by the single-cell local consistency for each RNA velocity method. d. Local consistency distributions for the three top and bottom cell types from the pancreas dataset, as ranked by average local consistency. (TIF) [file pcbi.1014303.s003.tif]

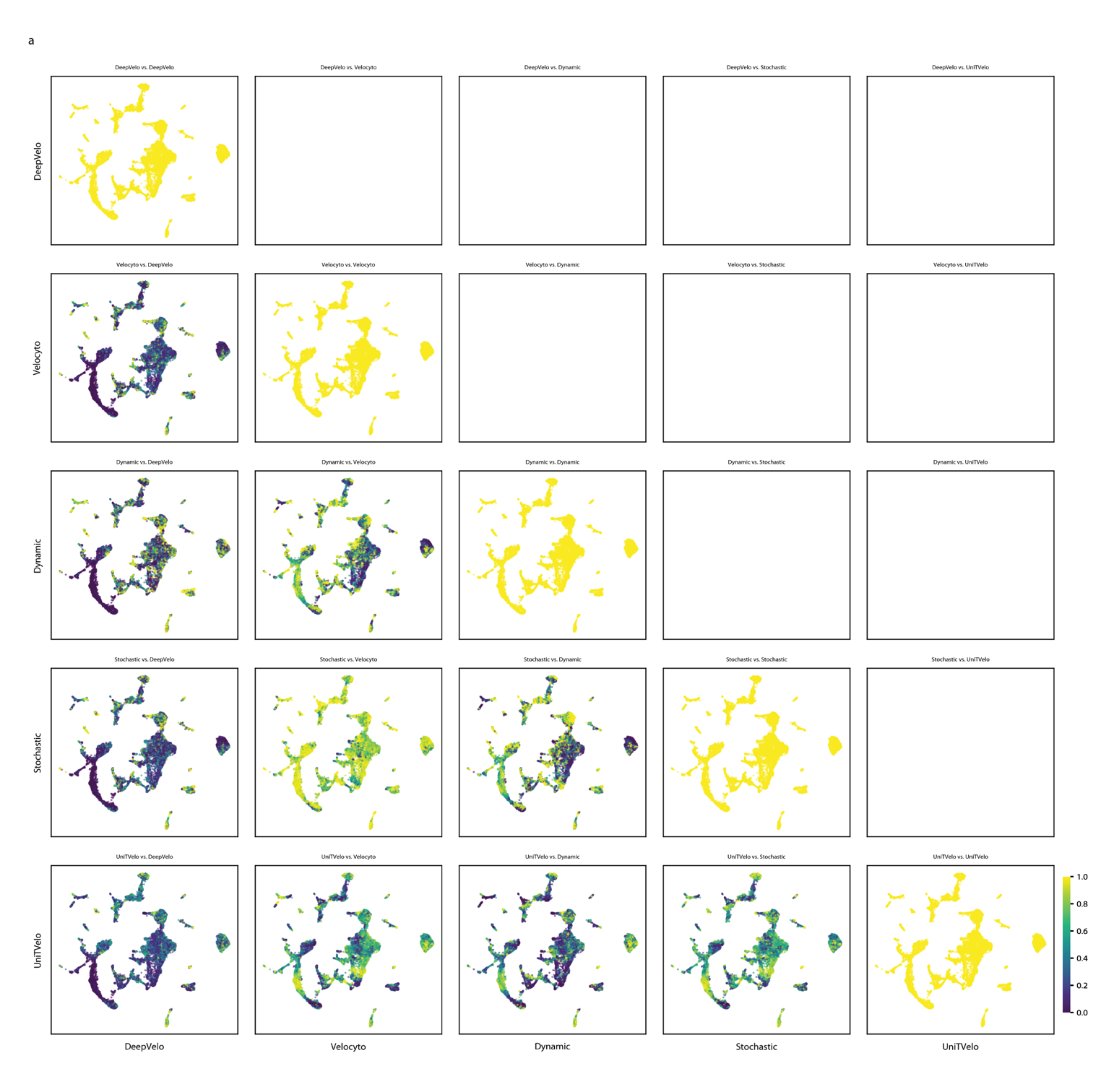

Supplement: S4 Fig — (TIF) [file pcbi.1014303.s004.tif]

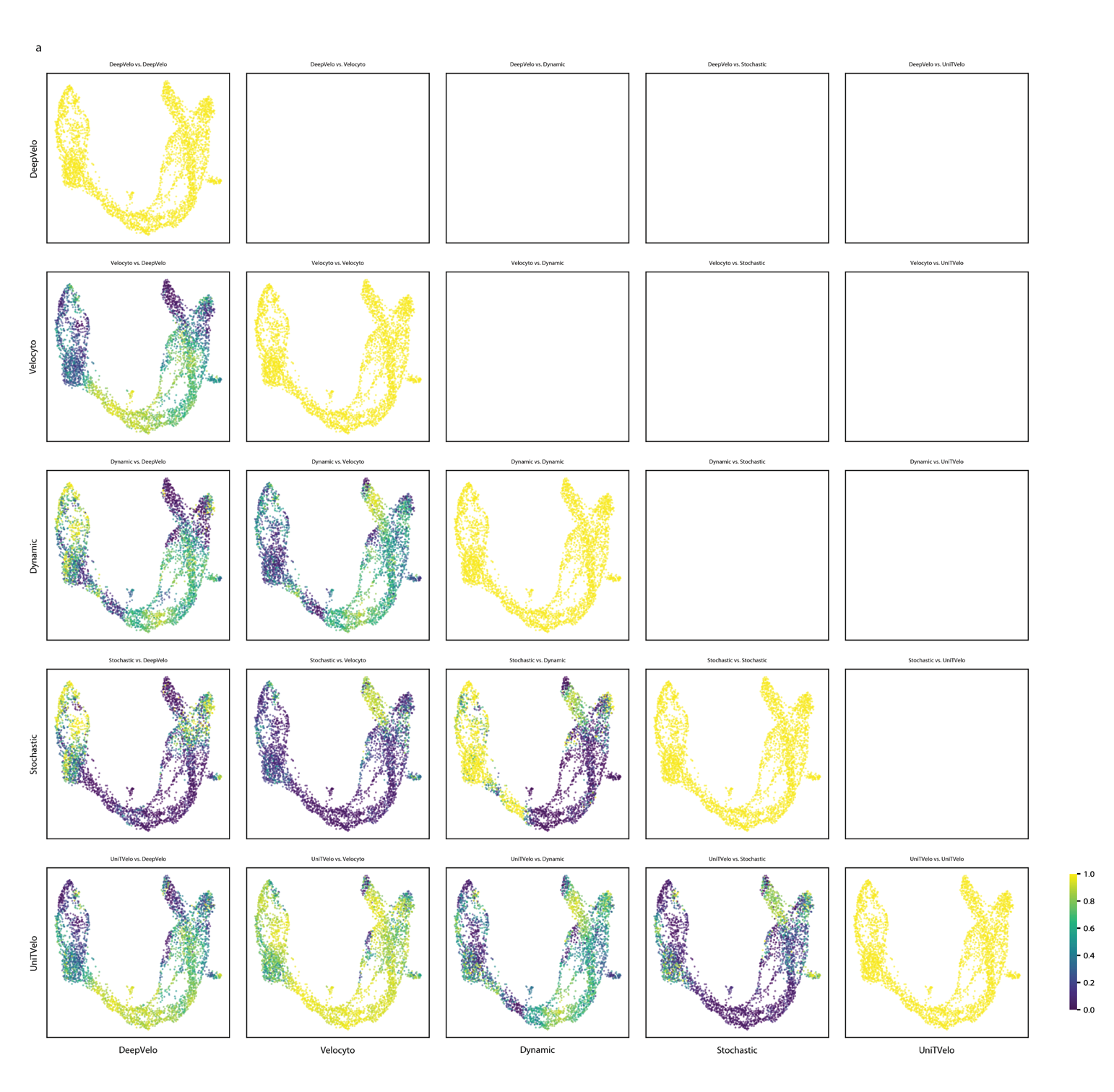

Supplement: S5 Fig — (TIF) [file pcbi.1014303.s005.tif]

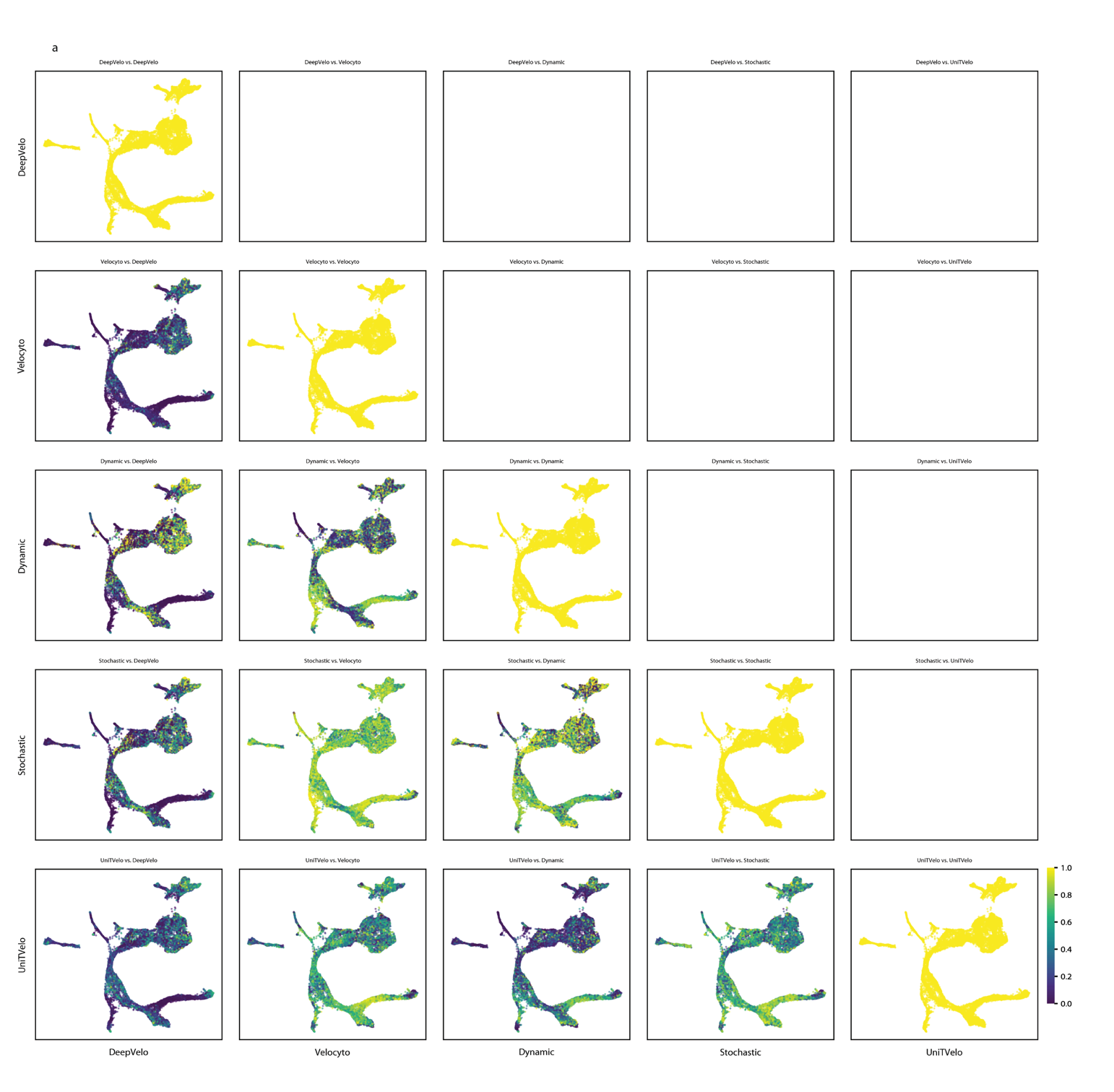

Supplement: S6 Fig — (TIF) [file pcbi.1014303.s006.tif]

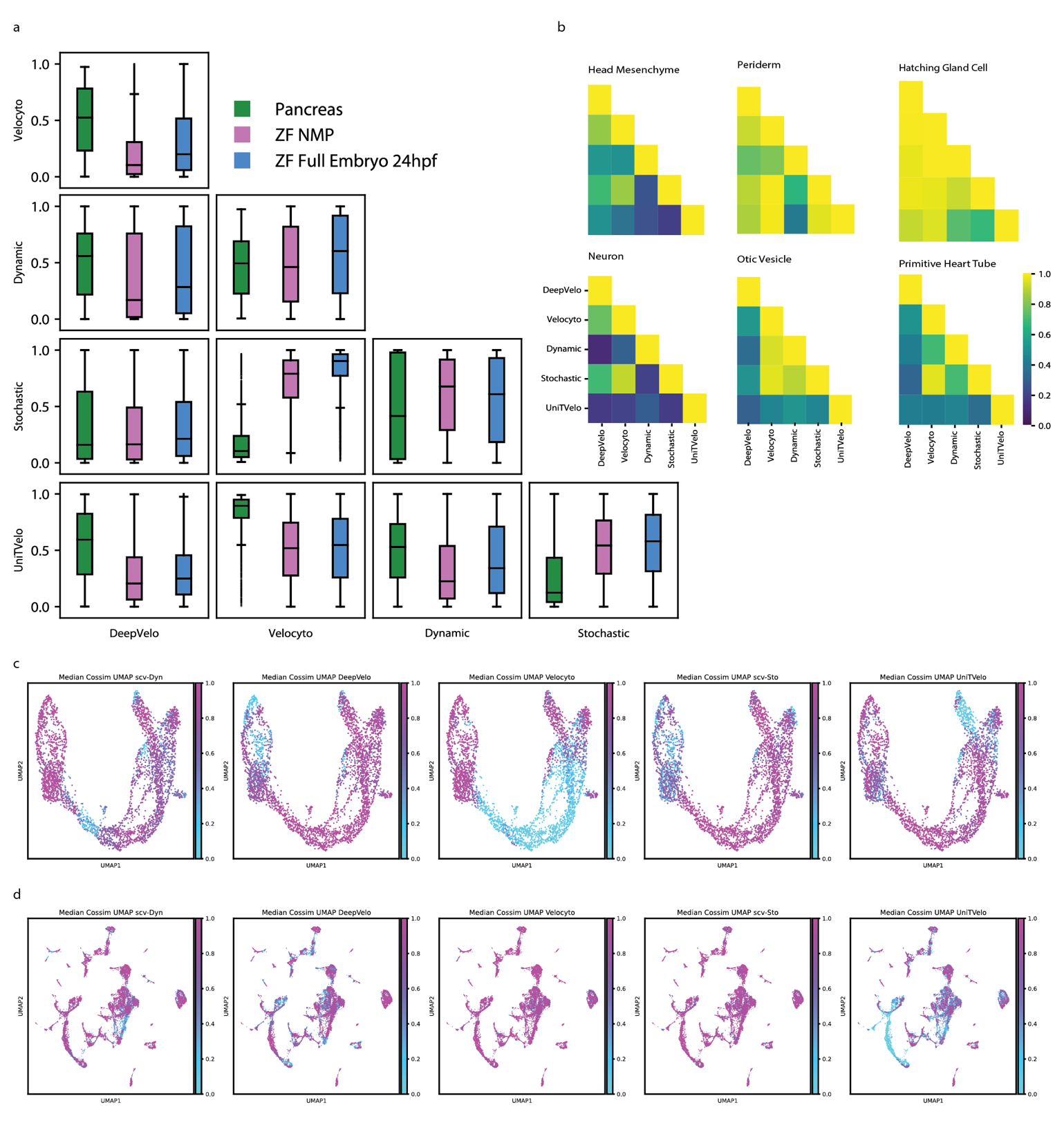

Supplement: S7 Fig — a. Boxplots with distributions of pairwise method agreement for each pair of method for each of the three datasets. b. Pairwise comparisons for six cell types from the ZF embryo 24hpf dataset across all methods. The heatmap shows the median method agreement across individual cells within a cell type for each pair of methods. c. UMAP embeddings for the pancreas for each RNA velocity method, colored by each method’s agreement (2) with the median vector. d. UMAP embeddings for ZF NMPs for each RNA velocity method, colored by each method’s agreement (2) with the median vector. (TIF) [file pcbi.1014303.s007.tif]

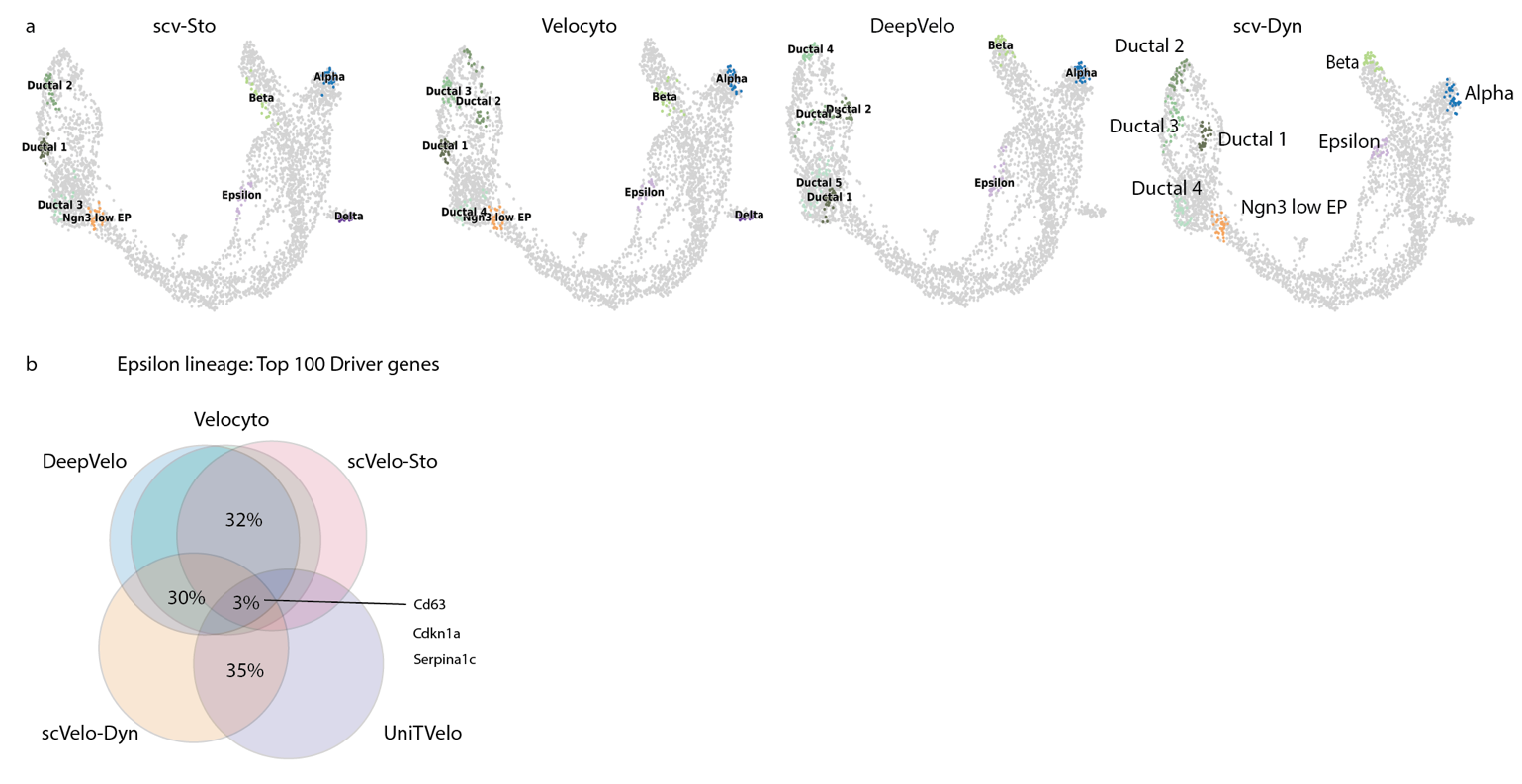

Supplement: S8 Fig — a. Macrostates identified by CellRank for scv-Sto, Velocyto, DeepVelo and scv-Dyn in the pancreas dataset. CellRank identified different macrostates for each dataset depending on the model’s predictions (see Methods). b. Venn diagram with the percentage overlap across all methods and select groups indicated of the top 100 driver genes for the Epsilon lineage. Genes identified across all methods as top driver genes are labeled. (TIF) [file pcbi.1014303.s008.tif]

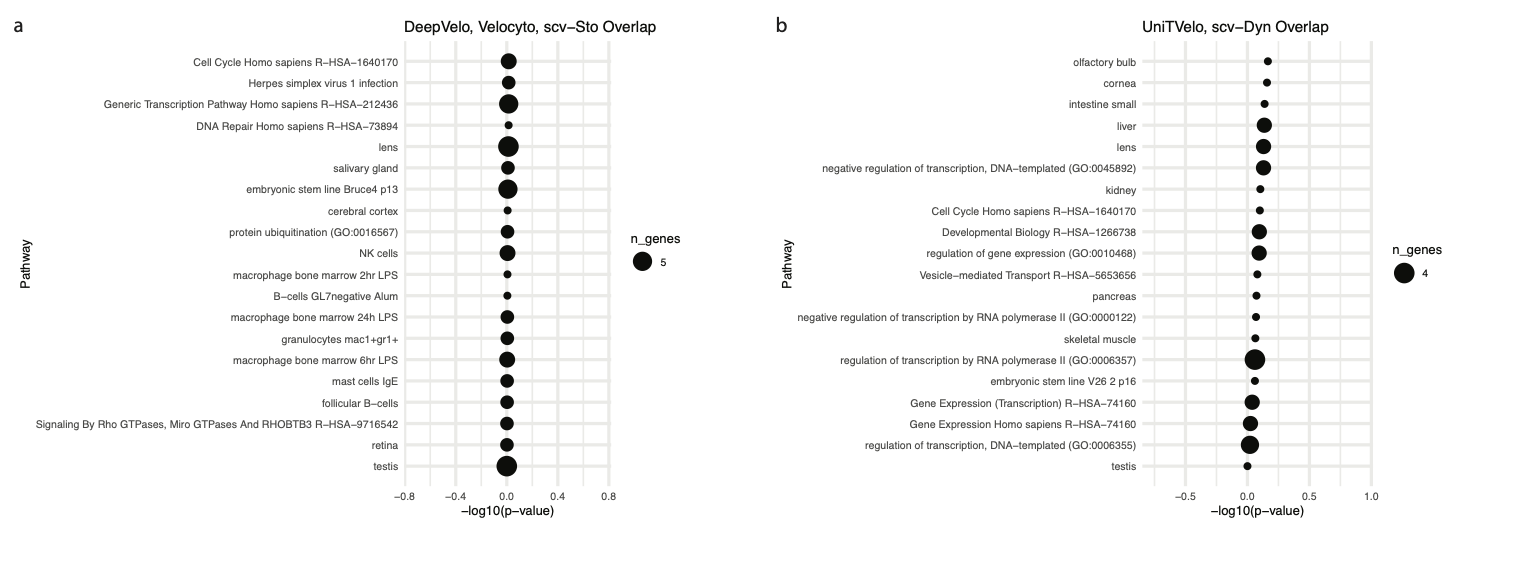

Supplement: S9 Fig — a. Gene ontology of the 79 genes overlapping for the beta lineage identified by DeepVelo, Velocyto, and scv-Sto. b. Gene ontology of the 55 genes overlapping for the beta lineage identified by UniTVelo and scv-Dyn. (TIF) [file pcbi.1014303.s009.tiff]

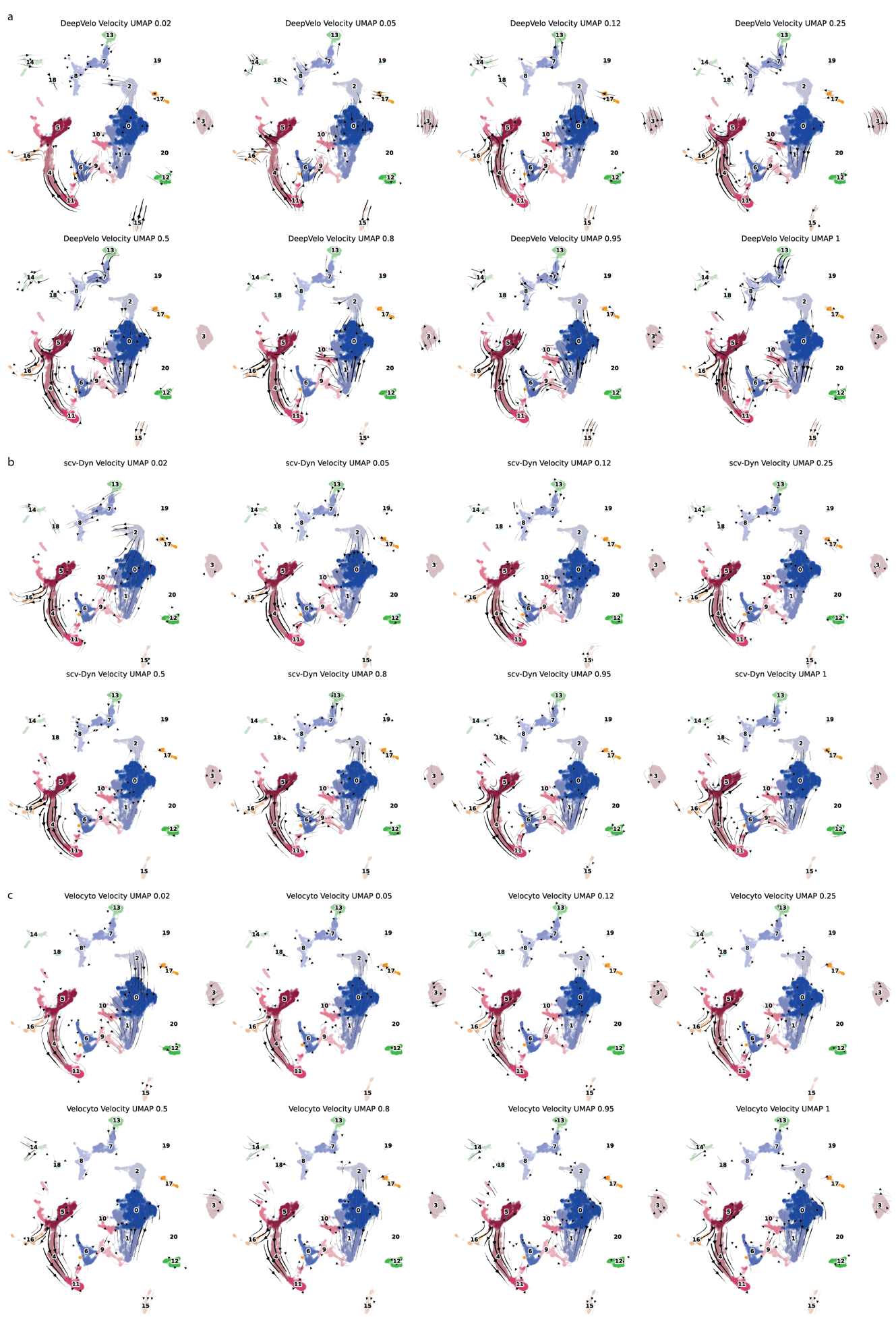

Supplement: S10 Fig — a. UMAP embeddings of the ZF 24hpf whole-embryo with RNA velocity predictions from DeepVelo, calculated from subsets 2, 5, 12, 25, 50, 80, 95 and 100% of the reads. b. UMAP embeddings of the ZF 24hpf whole-embryo with RNA velocity predictions from scv-Dyn, calculated from subsets 2, 5, 12, 25, 50, 80, 95 and 100% of the reads. c. UMAP embeddings of the ZF 24hpf whole-embryo with RNA velocity predictions from Velocyto, calculated from subsets 2, 5, 12, 25, 50, 80, 95 and 100% of the reads. (TIF) [file pcbi.1014303.s010.tif]

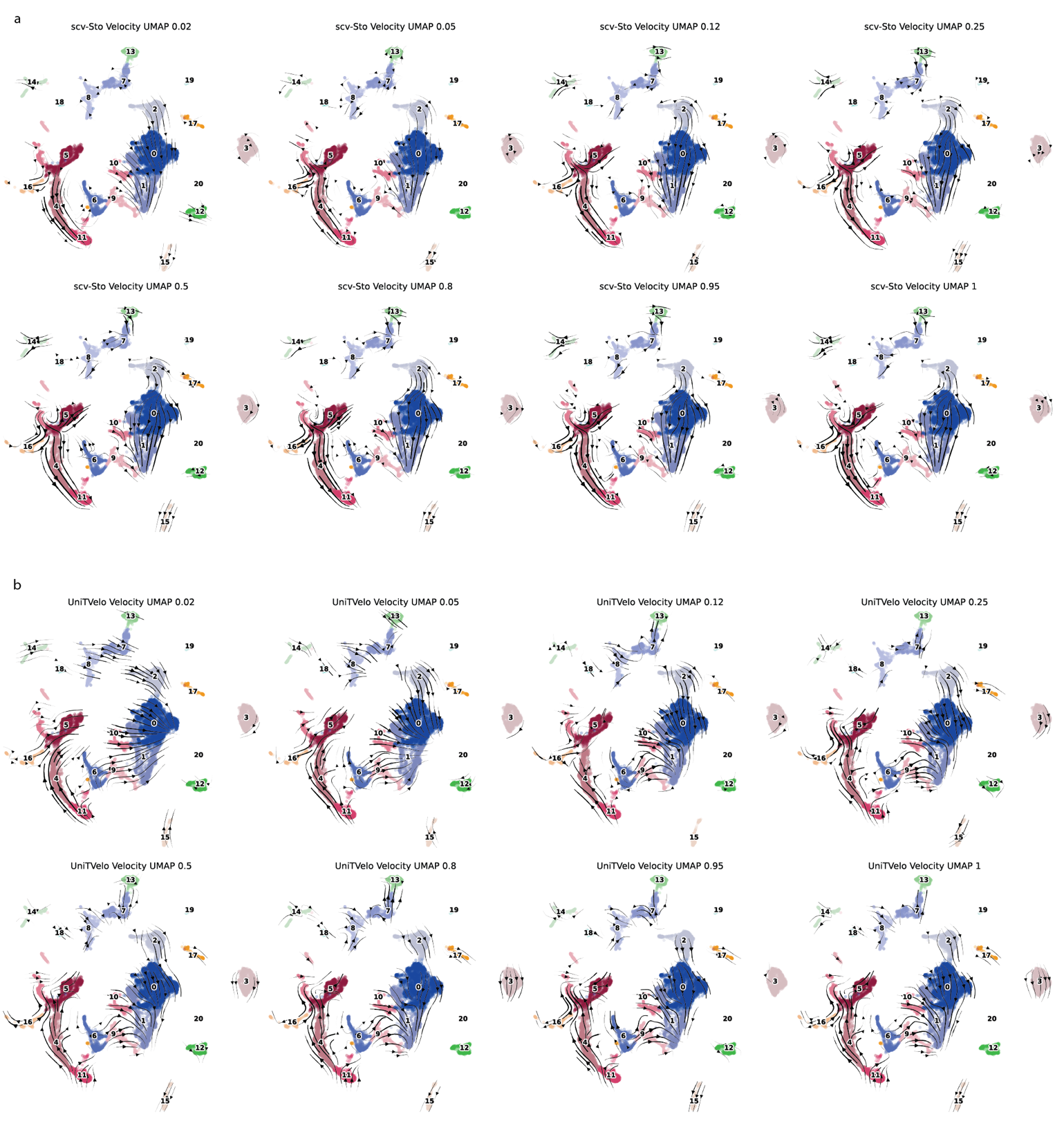

Supplement: S11 Fig — a. UMAP embeddings of the ZF 24hpf whole-embryo with RNA velocity predictions from scv-Sto calculated from subsets 2, 5, 12, 25, 50, 80, 95 and 100% of the reads. b. UMAP embeddings of the ZF 24hpf whole-embryo with RNA velocity predictions from UniTVelo, calculated from subsets 2, 5, 12, 25, 50, 80, 95 and 100% of the reads. (TIF) [file pcbi.1014303.s011.tif]

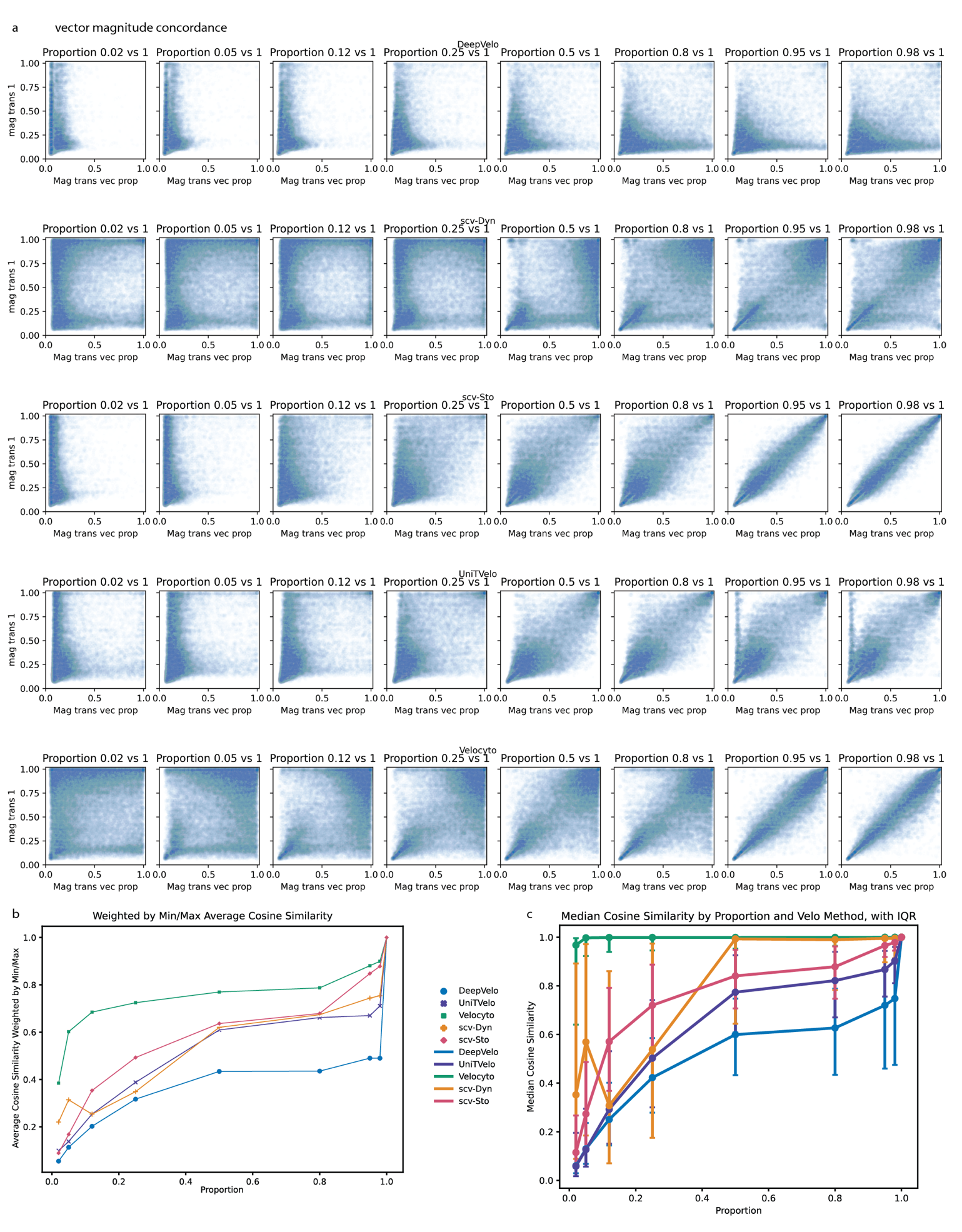

Supplement: S12 Fig — a. Scatterplots comparing the magnitude of the transition vector calculated from the subset reads (x-axis) vs. 100% of the reads (y-axis) for each method. The columns correspond to different subsets, with increasing proportions of reads (2, 5, 12, 25, 50, 80, 95, 98%), and the rows correspond to different methods (DeepVelo, scv-Dyn, scv-Sto, UniTVelo, Velocyto). b. Comparison of directionality robustness for each method weighted by the min/max, calculated as the cosine similarity of the transition vector from the subset with the directionality from the transition vector calculated with 100% of the reads. We multiply each similarity score by the minimum magnitude of the two vectors divided by the maximum magnitude. The line plot shows the averaged value across all cells and subset iterations in the ZF 24hpf whole-embryo dataset. c. Median and InterQuartile Range (IQR - 25% and 75% percentile) of the cosine similarity between the transition vector from the subset with the directionality from the transition vector calculated with 100% of the reads, calculated across all cells and subset iterations in the ZF embryo 24hpf dataset. (TIF) [file pcbi.1014303.s012.tif]

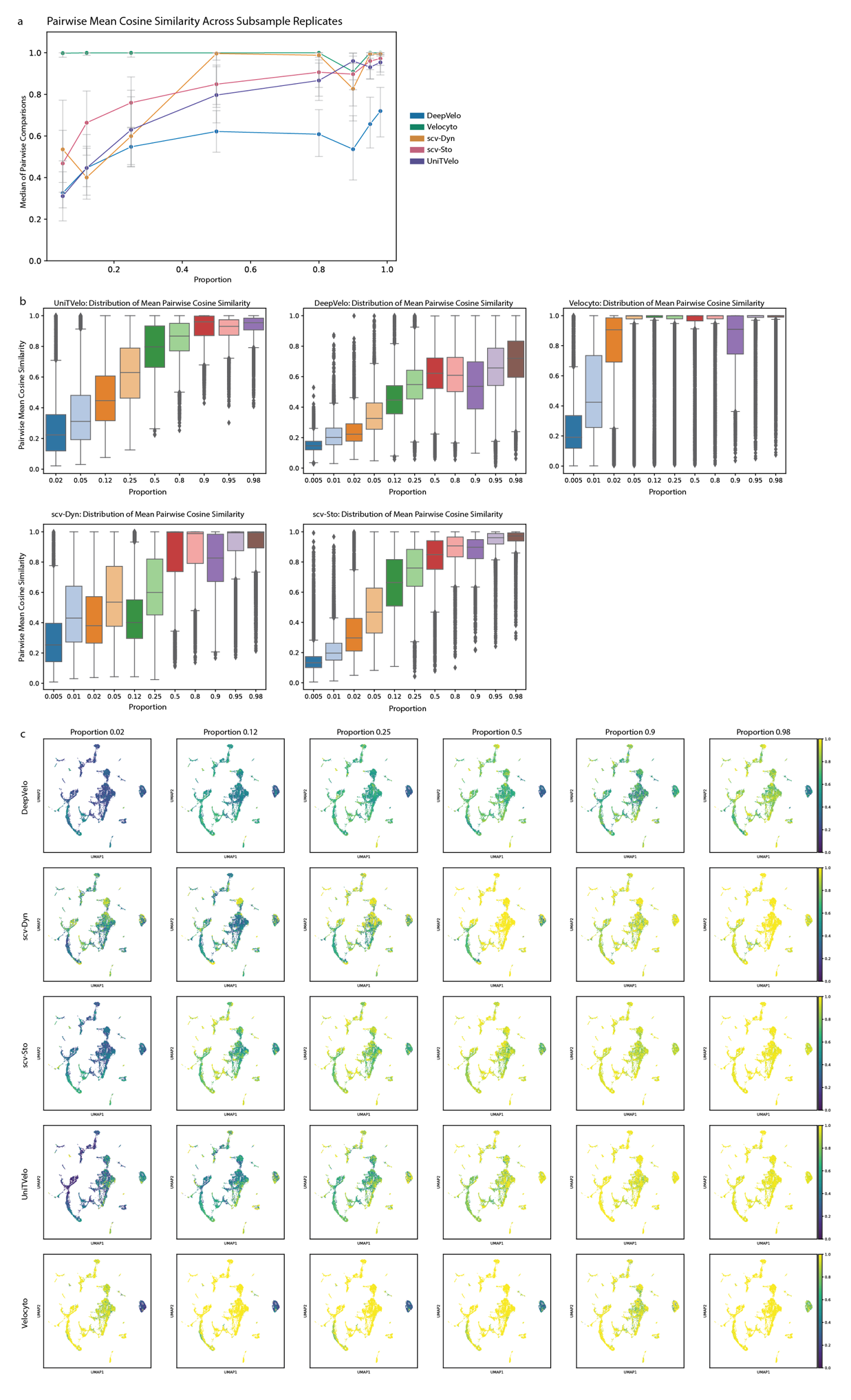

Supplement: S13 Fig — We plot the median and the 25% and 75% percentile (range indicated by the gray bars) of the mean pairwise cosine similarity scores across the ten unique replicate pairs of transition vectors for each cell and repeat for each method, at each proportion level of subset reads (5, 12, 25, 50, 80, 90, 95, 98%). b. Boxplot distribution of mean pairwise cosine similarity scores across proportion levels of subset reads for each method separately. c. UMAPs where each cell is colored by the mean pairwise cosine similarity of transition vectors across the ten unique pairs of replicates. The columns correspond to different subsets, with increasing proportions of reads (2, 12, 25, 50, 90, 98%), and the rows correspond to different methods (DeepVelo, scv-Dyn, scv-Sto, UniTVelo, Velocyto). (TIF) [file pcbi.1014303.s013.tif]
